# Brain Activity of Young Children in Bangladesh Is Associated With Biopsychosocial Conditions at the Individual‐, Family‐, and Household‐Level

**DOI:** 10.1111/infa.70108

**Published:** 2026-07-18

**Authors:** Leila M. Larson, Daniel Feuerriegel, Gitanjali Lall, Mohammed Imrul Hasan, Sabine Braat, Jerry Jin, Beverley‐Ann Biggs, Stefan Bode, Sant‐Rayn Pasricha, Katherine A. Johnson, Jena D. Hamadani

**Affiliations:** ^1^ Department of Health Promotion, Education, and Behavior, Arnold School of Public Health University of South Carolina Columbia South Carolina USA; ^2^ Melbourne School of Psychological Sciences The University of Melbourne Melbourne Victoria Australia; ^3^ Maternal and Child Health Division International Centre for Diarrhoeal Disease Research Dhaka Bangladesh; ^4^ Population Health and Immunity Division Walter and Eliza Hall Institute of Medical Research Melbourne Victoria Australia; ^5^ Centre for Epidemiology and Biostatistics, Melbourne School of Population and Global Health The University of Melbourne Melbourne Victoria Australia; ^6^ Department of Infectious Diseases at the Peter Doherty Institute The University of Melbourne Melbourne Victoria Australia; ^7^ The Victorian Infectious Diseases Service The Royal Melbourne Hospital Melbourne Victoria Australia; ^8^ Division of Science New York University Abu Dhabi Abu Dhabi UAE; ^9^ Diagnostic Haematology The Royal Melbourne Hospital Melbourne Victoria Australia; ^10^ Diagnostic Haematology and Clinical Haematology The Peter MacCallum Cancer Centre and The Royal Melbourne Hospital Melbourne Victoria Australia; ^11^ Department of Medical Biology The University of Melbourne Melbourne Victoria Australia

## Abstract

Adversities in early life compromise children's nurturing care and impact their development. This study explored the biopsychosocial conditions associated with electroencephalography (EEG)‐based brain activity in children living in rural Bangladesh, a setting with high rates of malnutrition and suboptimal psychosocial stimulation. In a substudy of the Benefits and Risks of Iron Supplementation in Children (BRISC) study in Bangladesh, we recorded resting EEG brain activity in a random subsample of 440 children at 11 months and 594 children at 20 months of age. EEG band power measures were derived for delta, theta, alpha, and beta power bands. Validated tools were used to assess maternal depression, household food insecurity, and psychosocial stimulation. Children's anthropometry, venous hemoglobin and ferritin concentrations, and inflammation were also measured. Linear regression analyses were conducted to identify the concurrent and lagged biopsychosocial conditions associated with EEG‐based brain activity. Growth, nutritional status, psychosocial stimulation, and household food security were associated with concurrent and lagged brain activity. The direction of association differed by EEG frequency band, but the majority of biopsychosocial conditions were positively associated with activity in the higher frequency bands and negatively with lower frequency bands. Our findings indicate that brain activity in children can be influenced by a range of biopsychosocial adversities. Children's nutrition, quality and quantity of psychosocial stimulation, and food security influence their current and later brain activity in this resource‐limited setting in South Asia.

**Trial Registration:**

ACTRN12617000660381

**Abbreviations**
CI: confidence interval
CRP: C‐reactive protein
EEG: electroencephalography
IQR: interquartile range
MNP: multiple micronutrient powder
RCT: randomized controlled trial
SD: standard deviation

## 1 Introduction

Approximately 36% of all children between 3 and 4 years of age in low‐ and middle‐income countries (LMIC) experience low cognitive and socioemotional development (McCoy et al. 2016), with 9.7% experiencing severe cognitive delays (Emerson and Llewellyn 2023). Early life adversity in the form of poverty, malnutrition, and suboptimal care impacts the overall development of children (Kirolos et al. 2022; Manalew et al. 2022). Few studies in resource‐limited settings, however, have examined the influence of a child's environment on their brain activity, an important outcome that has been shown to be predictive of later life cognitive and motor development (Braithwaite et al. 2020; Elansary et al. 2024; Jones et al. 2020; Xiao et al. 2017). Our study was based in rural Bangladesh, a setting with high rates of malnutrition and suboptimal psychosocial stimulation, which is defined as the family care practices and resources that promote the cognitive, language, motor and socio‐emotional development of children (Kariger et al. 2012). The study identified the biological and psychosocial (henceforth referred to as biopsychosocial) conditions associated with electroencephalography (EEG)‐based brain activity in young children.

Adverse experiences in early life in the form of suboptimal nurturing care have been shown to impact children's development, including long term impacts on brain structure and function (Miguel et al. 2019; Noble et al. 2015; Shonkoff et al. 2017). Studies using magnetic resonance imaging have shown differential patterns of activation of brain areas and reduced volume in certain brain areas in children exposed to a single severe adverse event or chronic threat and deprivation (McLaughlin et al. 2019; Noble et al. 2015). Similarly, the link between nutrition and brain development is well established, with research showing the impact of inadequate nutrition on the developing brain from conception through the first 1000 days (Cusick and Georgieff 2016; Ottolini et al. 2020; Prado and Dewey 2014). Various anatomical, chemical, physiological, and metabolic mechanisms explain the influence of micro and macronutrient status on neurodevelopment. For example, iron and zinc are essential micronutrients that support brain myelination and the development and function of neurotransmitters (Cusick and Georgieff 2016). The timing of exposure to adversity matters, with exposure to adversity during critical periods of brain development likely to have longer term impacts lasting into adulthood (Cusick and Georgieff 2016; Nelson and Gabard‐Durnam 2020; Ottolini et al. 2020; Prado and Dewey 2014).

Studies of EEG‐based brain activity are important given the growing understanding that it is a meaningful outcome in young children, predictive of later memory, cognitive, and language development (Brito et al. 2016; Gou et al. 2011; Nordvik et al. 2022; Xie et al. 2019). For instance, neonatal EEG gamma power has been associated with later childhood cognitive abilities related to declarative memory and auditory comprehension (Brito et al. 2016) as well as language abilities (Gou et al. 2011). EEG activity (as total absolute band powers of the delta, theta, alpha, and beta bands) in neonates has also been found to predict cognitive outcomes in late childhood (Nordvik et al. 2022).

Suboptimal environments in early life have been shown to impact EEG‐based brain activity (Harmony et al. 1988; Saby and Marshall 2012). For instance, in high chaos households, hearing more adult words has been associated with lower EEG band power at parietal electrodes (Brito et al. 2020). Differences in resting brain activity associated with language and working memory have been found in children growing up in low‐income compared to high‐income environments (Maguire and Schneider 2019), as well as those experiencing high compared low maternal stress (Peltola et al. 2014; Pierce et al. 2019; Pierce et al. 2021; Troller‐Renfree et al. 2023). EEG‐based brain activity has also been shown to be sensitive to nutritional status (Otero et al. 2019; Gustafson et al. 2022; Otero et al. 2019).

Most studies of early life EEG‐based brain activity are in children living in high‐income settings. Children in LMICs consistently face a host of important adversities, including malnutrition, repeated infection, and low psychosocial stimulation, and there is limited but emerging evidence showing that these adversities may be associated with EEG‐based brain activity. Emerging research has demonstrated the feasibility of conducting EEG measurements with limited resources in LMICs (Bhavnani et al. 2022; Chin et al. 2025; Larson et al. 2023). For instance, studies have shown that EEG‐based brain activity in children is associated with stimulation in the home environment in Mexico (Otero et al. 2003) and with cognitive development in India (Parameshwaran et al. 2025). Another study based in urban Bangladesh showed an association between low child linear growth, an indicator of a deprived environment, and EEG‐based brain functional connectivity (Xie et al. 2019). There is still, however, limited understanding of brain activity, and the biological and psychosocial influences on brain activity, in young children in these resource‐limited settings. Our study sought to identify the biopsychosocial conditions that are associated with concurrent and later EEG‐based brain activity in young children living in rural Bangladesh, a resource‐limited setting in South Asia.

## 2 Methods

Data were collected as part of a substudy nested within the Benefits and Risks of Iron Supplementation in Children (BRISC) trial (www.anzctr.org.au; [ACTRN12617000660381]). The BRISC study was a collaboration between the International Center for Diarrheal Disease Research, Bangladesh (icddr,b), the University of Melbourne, Australia, and the Walter and Eliza Hall Institute, Australia. The study was approved by the ethical review committees of icddr,b, and Melbourne Health.

### 2.1 Study Design

#### 2.1.1 BRISC Study Design

The BRISC study was a 3‐arm, double‐blind, double‐dummy, individual randomized, superiority trial conducted in the Rupganj Upazila of Narayanganj district, Bangladesh. A detailed explanation of the study characteristics is provided elsewhere (Pasricha et al. 2021). In brief, at 8 months of age +/− 14 days, 3300 children were randomized to receive 3 months of one of the following: (1) 12.5 mg elemental iron (ferrous sulfate) syrup + placebo multiple micronutrient powders (MNPs); (2) MNPs (containing iron 12.5 mg as ferrous fumarate, retinol 200 μg, zinc 5 mg, and vitamin C) + placebo iron syrup; or (3) placebo iron syrup and placebo MNPs. Exclusion criteria included severe anemia [hemoglobin < 80 g/L measured on HemoCue 301 (HemoCue) using a single‐drop capillary blood sample], drinking water iron content > 1 mg/L, mid ‐upper‐arm circumference < 11.5 cm, current illness with fever, or previously known inherited red cell disorders. Children were randomly assigned to one of the 3 arms using a 1:1:1 allocation, stratified by union (local area) of residence and sex. The study team and participants were blinded to the intervention. Assessments were completed in children at baseline, 3 months after intervention (month 3, when children were aged 11 months), and after a further 9‐month follow‐up (month 12, when children were aged 20 months). The study began recruitment in July 2017 and completed follow‐up visits in February 2020. The full BRISC study will henceforth be referred to as the parent study.

#### 2.1.2 Neurocognitive Substudy Design

Details of the substudy methods are described elsewhere (Larson et al. 2023). Briefly, starting in December 2018, using a random number generator, children enrolled in the parent trial were randomly included in the neurocognitive substudy starting at the postinterventional visit. Measurements for the neurocognitive substudy were conducted within 7 days after the measurements from the parent study immediately post‐intervention, when children were 11 months old, and at the 9‐month follow‐up visit, when the children were 20 months old. Children who completed an EEG recording at 11 months were asked to return for the EEG recording at 20 months of age. At the 9‐month follow up timepoint, when children were 20 months of age, additional children were randomly enrolled for EEG measurements. A total of 440 children were enrolled into the substudy immediately post‐intervention, of whom usable EEG data were available for 412 children. At the 9‐month follow up, 594 children were measured, of whom 374 provided usable EEG‐data.

### 2.2 Measurements

As part of the neurocognitive substudy, children's resting state EEG was measured in a dedicated testing room. Children, who were seated on their caregivers' lap, watched a video display consisting of 350 randomly generated Gabor patches superimposed on a green background. This display was used to maintain the child's attention throughout the recording. Although it involves more visual stimulation than typical resting EEG recordings in adults, it did not involve any type of cognitive task (similar to Stroganova et al. 1999; Tomalski et al. 2013). Caregivers were instructed to not talk to or distract their child. EEG‐based brain activity was recorded from 32 active scalp electrodes, placed according to the international 10–20 system Brainvision LiveAmp system (Brain Vision LLC) in line with standard procedures. EEG band power measures were derived from the original power spectra in the alpha (6–9 Hz) (mu and posterior), beta (9–30 Hz), theta (3–6 Hz) and delta (1–3 Hz) frequency bands (Larson et al. 2023). Theta and alpha bands were identified as the frequency bands that encompassed peaks in the Fourier spectra in both age groups, which were highly similar (spectra displayed in the Supporting Information S1 of Larson et al. 2023). EEG data preprocessing was conducted using EEGLab v13.4.4 and has been detailed in the Supporting Information S1. Briefly, data were first referenced to the average of electrodes TP9/10 for data collected at month 3. These electrodes were selected to approximate a linked mastoids reference. Data were instead referenced to electrode Pz for data collected at month 12, as the data quality at electrodes TP9/10 was too poor to use as a reference. We identified regions of interest (ROIs) that corresponded to relatively localized sources of frequency band‐limited activity on the scalp. This was done based on visual inspection of group average Fourier amplitude spectra, prior to unblinding of treatment condition and the participant variables used for our analyses (spectra displayed in the Supporting Information S1 of Larson et al. 2023). These included electrode Fz (frontal ROI) for delta, electrodes Fz, F3, F4, FC1, FC2, Cz, C3, C4 (frontocentral ROI) for theta, and two distinct ROIs for alpha: Oz, O1, O2 (posterior alpha ROI) and Cz, C3, C4 (central/mu alpha ROI). Clear localization could not be determined for beta activity based on observation of the scalp maps and we did not have any a priori reasons for assuming a localized source, therefore the average of electrodes Fz, F3, F4, FC1, FC2, Cz, C3, C4, CP1, CP2, Pz, P3, P4, Oz, O1, O2 was used for measures of activity in this frequency band. The mean power across each frequency band, averaged across channels in each ROI was calculated, obtaining one value per participant.

As part of the parent trial, children's biological and psychosocial characteristics were measured at 8, 11, and 20 months of age (Pasricha et al. 2021). Children's anthropometry was taken and growth indices of length‐for‐age, weight‐for‐age, and weight‐for‐height *Z*‐scores were derived using the World Health Organization 2006 growth standards. Venous hemoglobin concentration was measured in g/L using a HemoCue 301+ device (HemoCue). Serum ferritin was measured in g/mL by electrochemiluminescence immunoassay using the Cobas e601 analyzer (Roche Diagnostics) and C‐reactive protein (CRP) in mg/L by particle‐enhanced immunoturbidimetric assay using the Cobas c311 analyzer (Roche Diagnostics).

Maternal depression was measured using the Center for Epidemiological Studies‐Depression (CES‐D) scale (Radloff 1977), a measure that asks caregivers to rate how often they have experienced symptoms associated with depression over the past week. Scores range from 0 to 35, with higher scores indicating a higher level of depression. Household food insecurity was measured using the Household Food Insecurity Access Scale (HFIAS) (Coates et al. 2007). Higher scores are indicative of higher food insecurity, and participants were categorized as being food secure or food insecure. Psychosocial stimulation in the home environment of children was measured using the Family Care Indicators (FCI) (Kariger et al. 2012). Higher scores on the FCI are indicative of higher stimulation. All these measures have been validated for use across a range of countries and settings, including Bangladesh (Black et al. 2007, 2009; Frongillo 2022; Frongillo et al. 2003; Hamadani et al. 2010; Nahar et al. 2015; Swindale and Bilinsky 2006).

### 2.3 Sample Size

Our analysis included all children for whom EEG data were available at any timepoint (i.e., 11 months or 20 months child's age). The sample size was informed by the neurocognitive substudy's primary outcome of resting state EEG and based on a meaningful standardized difference between intervention and control group of 0.4, with 80% power at a 2‐sided 5% alpha level. Specifically, artifact free EEG data was obtained for 412 children at 11 months of age and 374 children at 20 months of age, including 257 newly recruited children. A total of 103 children who underwent assessment at 11 months did not receive an EEG measurement at 20 months owing to loss to follow‐up or parents declining a follow‐up measurement (Larson et al. 2023).

### 2.4 Statistical Analysis

We selected biopsychosocial conditions as independent variables because of their plausible influence on EEG outcomes based on previous literature. These included maternal depression, food insecurity, psychosocial stimulation, hemoglobin (g/L), ferritin (g/mL), C‐reactive protein (mg/L), weight‐for‐age *z*‐scores, and length‐for‐age *z*‐scores. Weight‐for‐height *z*‐scores were not included due to being highly correlated with weight‐for‐age and length‐for‐age *z*‐scores.

Descriptive data were presented as frequency count and percentage (%) for categorical variables, mean and standard deviation (SD) for normally distributed continuous variables, and median and quartiles (25th and 75th) for non‐normally distributed variables. Ferritin and CRP were log_10_‐transformed due to their positive skewness.

We conducted linear regression analyses to examine associations between each biopsychosocial condition and each of the EEG band power measures, both in the absence (referred to as “unadjusted”) and presence (referred to as “adjusted”) of the other biopsychosocial conditions (Supporting Information S1: Tables 1–5). We examined concurrent and lagged associations between biopsychosocial conditions at 8, 11, and 20 months of age and EEG‐based brain activity at 11 and 20 months of age, resulting in five exposure‐outcome settings for each EEG frequency band. Model 1 examined concurrent associations between biopsychosocial conditions and EEG outcomes measured at 11 months of age; Model 2 examined concurrent associations between biopsychosocial conditions and EEG outcomes measured at 20 months of age; Model 3 examined lagged associations between biopsychosocial conditions measured at 8 months of age and EEG outcomes measured at 11 months of age; Model 4 examined lagged associations between biopsychosocial conditions measured at 8 months of age and EEG outcomes measured at 20 months of age; Model 5 examined lagged associations between biopsychosocial conditions measured at 11 months of age and EEG outcomes measured at 20 months of age. Underlying assumptions of the linear regression model were checked, namely normality of residuals, homoscedasticity, and linearity of relationship between independent and dependent variable. Variables not found to be normally distributed were log transformed. All other assumptions were met. A false discovery rate (FDR) correction procedure (at *Q* = 0.05) was applied to all models to adjust for multiple testing (Benjamini and Hochberg 1995).

All analyses accounted for the number of children under 5 years of age living in the household, parental education (number of years of schooling), wealth index (quintiles from relative poorest to relative wealthiest), religion (Islam or other), child sex (female or male), union (Bhulta, Golakandail, or Rupganj), and intervention group assignment (iron syrup, MNP, or placebo) collected when the child was 8 months of age. Participants who had missing values on any variables included in the analysis were removed from the analyses. We obtained the estimates of association, two‐sided 95% confidence intervals (CI), and two‐sided *p*‐values. Standardized estimates were presented as Cohen's d and 95% CI.

Analyses were performed using R version 4.4.2 (2024‐10‐31) (R Foundation for Statistical Computing, Vienna, Austria).

## 3 Results

Of the children assessed at month 3, 50.2% were female, 19.6% came from food insecure households, 26.0% were stunted, and 43.9% were anemic. Of the children assessed at month 12, 52.1% were female, 17.1% came from food insecure households, 23.5% were stunted and 48.6% were anemic (Table 1).

**TABLE 1:** Participant characteristics at baseline^a^.

	Children with sufficient artifact‐free EEG data at 11 months of age *N* = 412	Children with sufficient artifact‐free EEG data at 20 months of age *N* = 374
Household characteristics		
Union		
Bhulta	136/412 (33.0%)	125/374 (33.4%)
Golakandail	143/412 (34.7%)	125/374 (33.4%)
Rupganj	133/412 (32.3%)	124/374 (33.2%)
Religion		
Islam	382/412 (92.7%)	344/374 (92.0%)
Other (Hindu, Buddhism, Christian)	30/412 (7.3%)	30/374 (8.0%)
Number of children under 5 years of age living in the household	2 (1–3)	2 (1–3)
Maternal education (years)	2 (2–3)	2 (2–3)
Paternal education (years)	2 (2–3)	2 (2–3)
Wealth index		
Quintile 1 (relative poorest)	92/412 (22.3%)	96/374 (25.7%)
Quintile 2	76/412 (18.4%)	83/374 (22.2%)
Quintile 3 (relative middle)	99/412 (24.0%)	73/374 (19.5%)
Quintile 4	66/412 (16.0%)	59/374 (15.8%)
Quintile 5 (relative wealthiest)	79/412 (19.2%)	63/374 (16.8%)
Maternal depression score^b^	5 (1–10)	5 (1–12)
Household food secure status^c^	328/408 (80.4%)	306/369 (82.9%)
Child characteristics		
Female sex	207/412 (50.2%)	195/374 (52.1%)
Age (months)	8.0 (0.3)	8.0 (0.3)
Family care indicator total score^d^	13.2 (7.1)	13.1 (7.1)
Hemoglobin concentration (g/L) venous	110.0 (9.7)	109.3 (9.0)
Anemic (venous hemoglobin concentration < 110 g/L)	175/399 (43.9%)	175/360 (48.6%)
Ferritin (ug/L)	23.6 (13.4–38.1)	22.8 (11.8–38.5)
C‐reactive protein (mg/L)	0.84 (0.35–2.86)	0.82 (0.35–2.53)
Length/height‐for‐age *z*‐score	−1.36 (1.04)	−1.29 (1.02)
Stunted (length/height‐for‐age *z*‐score < −2)	106/408 (26.0%)	87/370 (23.5%)
Weight‐for‐age *z*‐score	−0.57 (1.04)	−0.60 (0.99)
Weight‐for‐length/height *z*‐score	0.35 (1.02)	0.26 (1.02)

^a^ Data are presented as mean (SD) or median (IQR) for continuous measures, and *n*/total (%) for categorical measures. Percentages may not total 100 because of rounding. EEG, electroencephalography.

^b^ Scores on maternal depression range from 0 to 35, with higher scores indicating more depressive symptoms.

^c^ Food secure was defined “no” or “rarely” to question 1 and “no” to questions 2–9 on the Household Food Insecurity Assess Scale.

^d^ Scores on family care indicator (psychosocial stimulation) ranged from 0 to 42, with higher scores indicating more activities/use of play or reading material.

**FIGURE 1:**
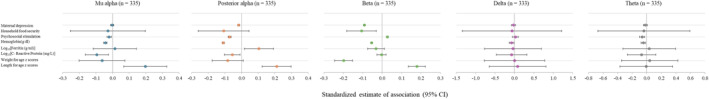
Standardized estimates (95% CI) for the concurrent associations between EEG bands and psychosocial conditions measured at 11 months child's age^1^. ^1^Maternal depression was measured using Center for Epidemiological Studies‐Depression scale (score range 0–35). Household food security was measured using the Household Food Insecurity Access Scale (scored as food secure or not). Psychosocial stimulation was measured using the Family Care Indicators (score range 0–42). Results were obtained from linear regression models which included all biopsychosocial characteristics, adjusted for number of children under 5 years of age living in the household, parental education, wealth, child sex, union, and intervention group assignment. The standardized estimate of association (95% CI) between maternal depression and posterior alpha was −0.017 (−0.024, −0.010), between maternal depression and beta was −0.091 (−0.094, −0.088), between psychosocial stimulation and beta was −0.071 (−0.081, −0.062), and between hemoglobin concentration and beta was −0.11 (−0.117, −0.103). CI = Confidence Interval, EEG = electroencephalography.

**FIGURE 2:**
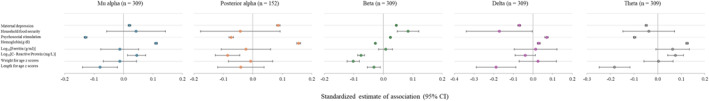
Standardized estimates (95% CI) for the concurrent associations between EEG bands and psychosocial conditions measured at 20 months child's age^1^. ^1^Maternal depression was measured using Center for Epidemiological Studies‐Depression scale (score range 0–35). Household food security was measured using the Household Food Insecurity Access Scale (scored as food secure or not). Psychosocial stimulation was measured using the Family Care Indicators (score range 0–42). Results were obtained from linear regression models which included all biopsychosocial characteristics, adjusted for number of children under 5 years of age living in the household, parental education, wealth, child sex, union, and intervention group assignment. The standardized estimate of association (95% CI) between maternal depression and beta was 0.044 (0.042, 0.046), between psychosocial stimulation and beta was 0.024 (0.022, 0.026), between hemoglobin concentration and beta was −0.027 (−0.029, −0.025), between maternal depression and theta was −0.049 (−0.054, −0.044), between psychosocial stimulation and theta was −0.098(−0.104, −0.092), and between hemoglobin concentration and theta was 0.125 (0.119, 0.130). CI = Confidence Interval, EEG = electroencephalography.

The biopsychosocial conditions associated with EEG outcomes varied by EEG band power and timing of measurement (Figures 1, 2). The results below describe uncorrected associations; After adjusting for multiple comparisons, only the concurrent association between household food insecurity and delta band power at 20 months remained significant.

Children's growth and nutritional status were associated with concurrent and later EEG power across different frequency bands. Concurrent analyses indicated that linear growth of children at 11 months of age was associated with their brain activity. Length‐for‐age *z*‐scores of children at 11 months were associated with concurrent mu alpha [Standardized *β* = 0.197 (95% CI: 0.068, 0.326)], posterior alpha [0.211 (0.123, 0.299)] and beta [0.179 (0.135, 0.222)] power. Higher length‐for‐age *z*‐scores at 20 months of age were associated with concurrently lower activity in the delta [−0.187 (−0.288, −0.087)] and theta [−0.183 (−0.248, −0.117)] bands. Lagged analyses indicated that length‐for‐age *z*‐scores of children at 8 months were positively associated with mu alpha power at 11 months [0.155 (0.044, 0.266)]. Weight‐for‐age *z* scores were found to be associated with concurrent brain activity, with higher weight‐for‐age *z* scores at 11 months of age being associated with lower brain activity in the beta band [−0.198 (−0.245, −0.152)]. Lagged analyses indicated that children's ferritin concentration at 8 months of age was associated with lower later delta band power at 20 months [−0.243 (−0.396, 0.091)] (Supporting Information S1: Tables 1–5).

Household food security status and psychosocial stimulation were associated with concurrent and later brain activity (Figures 1 and 2). Higher household food security at 20 months was associated with concurrently lower delta band power [−0.171 (−0.340, − 0.003)]. Psychosocial stimulation scores were negatively associated with concurrent and lagged EEG outcomes. Higher psychosocial stimulation at 20 months of age was associated with concurrently lower mu alpha power [−0.129 (−0.134, −0.124)]. Higher psychosocial stimulation at 8 and 11 months of age was associated with lower beta band power [−0.192 (−0.202, −0.182)] at 20 months and lower theta band power at 20 months [−0.140 (−0.143, −0.138)] (Supporting Information S1: Tables 1–5).

## 4 Discussion

In rural Bangladesh, young children's resting state brain activity was associated with concurrent (at 11 months or 20 months of age) measures of child growth, household food insecurity, and psychosocial stimulation, and with earlier (3–12 months prior) measures of growth, psychosocial stimulation, and iron status. Directions and magnitudes of associations varied by EEG frequency band. Concurrent linear growth was associated with lower power in the delta and theta bands, and higher power in the alpha (mu and posterior) and beta bands, resembling a flattening of the fourier power spectrum that has been observed to occur with increased age (Hill et al. 2022). Mu alpha was also positively associated with prior (i.e., 3 months earlier) linear growth. A different pattern of associations was observed for psychosocial stimulation, with concurrent psychosocial stimulation associated with lower mu alpha power at 20 months, and previous psychosocial stimulation associated with lower theta and beta power. In addition, delta band power at 20 months of age was negatively associated with concurrent food security and prior ferritin concentration. Beta power was negatively associated with weight‐for‐age *z*‐scores at 11 months of age. These findings provide further evidence that biopsychosocial conditions during early life influence brain activity in young children.

### 4.1 Nutritional Status

Research has shown that, as typically developing children age, EEG activity increases in the alpha and beta power bands and decreases in the delta and theta power bands (Cellier et al. 2021; Hill et al. 2022; Matousek and Petersen 1973). These changes reflect a “flattening” of the power spectrum curve with age, where the distribution of relative power shifts from lower to higher frequency ranges with maturation. Our study showed spectral changes associated with linear growth during early childhood, with higher linear growth being associated with increased activity in the alpha and beta power bands. These findings reinforce that improvements in growth, a proxy for a child's nutritional and morbidity status, co‐occur with changes in patterns of functional brain activity.

Our analysis showed that higher household food security and higher ferritin concentrations were associated with a lower delta band power. This is generally congruent with observations that power in low frequency bands reduces throughout the course of development. Research on the delta frequency band in early life, however, is limited, with our study being one of the first to report associations between biopsychosocial conditions and delta band power in young children.

### 4.2 Psychosocial Stimulation

With respect to responsive care and early learning opportunities, our study indicated that the quality and quantity of psychosocial stimulation in the child's home was negatively associated with concurrent brain activity (i.e., beta band power) and later brain activity (i.e., theta band power). This is in line with previous research showing negative associations between improved psychosocial conditions and theta and beta band power (Pierce et al. 2021; Troller‐Renfree et al. 2023; Xie et al. 2019). The negative association between psychosocial stimulation and mu alpha band power was unexpected given mu alpha typically increases with maturation (Marshall et al. 2002; Perego et al. 2016) and has been associated with higher levels of home stimulation in previous studies (Otero et al. 2003). Research has also shown the effects of improving psychosocial deprivation on alpha activity (Peltola et al. 2014; Perego et al. 2016; Vanderwert et al. 2010). For instance, in a study of Romanian children, removal of psychosocial deprivation in institutionalized children was associated with increases in high frequency alpha activity in the brain (Vanderwert et al. 2010). Few studies, however, have examined EEG‐based brain activity and its association with the quality and quantity of home stimulation within LMIC settings. The small but negative association with mu alpha in our study could be a function of the study setting and population, which had relatively low levels of psychosocial stimulation that are typical for resource limited settings (Lu et al. 2016; McCoy et al. 2016). This finding, however, merits further investigation.

### 4.3 Contextual Differences

Our results suggest that context may play a role in how biopsychosocial conditions are related to brain activity in young children. Studies in high income countries have examined associations between measures of adversity and EEG‐based brain activity in infants and young children with partial agreement with our results. For instance, higher maternal stress in low‐income families in the United States has been associated with higher theta and beta power and lower alpha and gamma activity in infants (Pierce et al. 2021; Troller‐Renfree et al. 2023). Greater numbers of vocalizations and conversational turns, which are indicative of language development, have been associated with lower relative theta power and higher relative gamma power (Pierce et al. 2021). Living in chaotic and disorganized households has also been correlated with lower activity in the beta band (Brito et al. 2020). Our findings that contrast with prior literature could be explained by the distinct study population experiencing a combination of complex adversities which may be differently associated with patterns of brain activity.

### 4.4 Strengths and Limitations

The nurturing care framework establishes the importance of optimizing children's health, nutrition, responsive caregiving, early learning, and safety and security to enable children to thrive (UNICEF World Bank and World Health Organization 2018). Our study is one of the first to explore how these conditions are associated with brain activity in young children living in a rural low‐income setting in South Asia. Our findings reinforce the association between linear growth and brain development, which has a strong foundation in the developmental literature using behavioral and self‐report measures (Perkins et al. 2017). Our study adds to this body of work by demonstrating that linear growth, a measure of a child's deprived environment, is associated with concurrent and later EEG‐based brain activity in the alpha, beta, delta and theta bands. These findings are meaningful given brain activity in the mu alpha band is associated with and predictive of motor development (Xiao et al. 2017), activity in the beta, theta and gamma bands is associated with language (Brito et al. 2016; Pierce et al. 2021), and activity in the beta and theta bands is associated cognitive development (Xie et al. 2019). Our findings are in line with previous research in Bangladesh, which found an association between linear growth and brain activity in the beta and theta bands in 48 month‐old children (Xie et al. 2019).

Our study benefited from a large sample size, particularly for a pediatric EEG research study. The sample size was reduced because of missing data on ferritin concentration and CRP concentration due to insufficient venous blood samples, as well as missing EEG data due to data loss from substantial artifacts in the data. However, EEG data loss was typical for EEG studies in children. After adjusting for multiple comparisons, many of the associations were no longer significant. Given, however, the exploratory nature of this analysis and the novelty of the study design for the setting, the unadjusted results provide an important view of how biopsychosocial conditions relate to early brain development in rural Bangladesh. While our study elucidates the relationship between brain activity and many individual, family, and household levels conditions, there are many other factors that might impact cognitive development that were not measured as part of our study. Similarly, our measurements are conceptually relatively broad, and do not reflect all detailed aspects of what constitutes the children's environment, family, household, and other relevant experiences.

## 5 Conclusions

While the literature on influences of biopsychosocial conditions on brain activity among young children in resource‐limited settings is emerging, our findings support a comprehensive approach to address the multiple adversities that children face in these settings to improve neurocognition. Cash transfer programs, for instance, have been shown to impact brain activity of young children from low‐income families (Troller‐Renfree et al. 2022), likely because these types of programs address multiple components of the nurturing care framework. Our results also point toward complex associations between co‐occurring and interconnected adversities and brain activity in young infants. How certain biological, environmental, and psychosocial conditions that influence brain activity may differ based on other existing conditions that can impede or enhance their influence. For instance, quality and quantity of responsive stimulation in the home have been shown to moderate the benefits of enhanced nutrition on neurodevelopment of children; children receiving better psychosocial stimulation benefit more from improved nutrition in resource poor settings (Larson et al. 2018). Importantly, this literature base establishes the modifiable nature of EEG‐based brain activity in response to intervention.

Our study demonstrated the importance of children's biopsychosocial conditions, namely nutrition, psychosocial stimulation, and food insecurity, on their concurrent and later brain activity in a rural low‐income setting in the Global South. Findings reinforce the critical nature of the nurturing care framework for enabling children to thrive, including through brain activity. Future research is needed from low‐income settings globally to understand how different forms of adversity in early life impact brain activity of young children and how these outcomes are associated with functional health and developmental outcomes later in life.

## Author Contributions

**Leila M. Larson:** conceptualization, methodology, formal analysis, supervision, funding acquisition, writing – original draft, investigation. **Daniel Feuerriegel:** conceptualization, methodology, data curation, investigation, supervision, writing – original draft. **Gitanjali Lall:** methodology, formal analysis, visualization, writing – original draft. **Mohammed Imrul Hasan:** investigation, project administration, writing – review and editing. **Sabine Braat:** conceptualization, methodology, formal analysis, writing – review and editing, visualization. **Jerry Jin:** data curation, formal analysis, writing – review and editing. **Beverley‐Ann Biggs:** conceptualization, supervision, writing – review and editing, funding acquisition. **Stefan Bode:** conceptualization, supervision, writing – review and editing. **Sant‐Rayn Pasricha:** conceptualization, methodology, supervision, writing – review and editing, funding acquisition. **Katherine A. Johnson:** conceptualization, supervision, writing – review and editing. **Jena D. Hamadani:** conceptualization, methodology, supervision, funding acquisition, writing – review and editing.

## Funding

This study was supported by grants (GNT1103262, GNT1159151, and GNT1158696) from the National Health and Medical Research Council of Australia, and by the University of Melbourne.

## Conflicts of Interest

The authors declare no conflicts of interest.

## Supporting information


Supporting Information S1

## Data Availability

De‐identified individual participant data that support the findings of this study are available from the corresponding author upon reasonable request.
