# Supplementary material for: Brain Activity of Young Children in Bangladesh Is Associated With Biopsychosocial Conditions at the Individual‐, Family‐, and Household‐Level
Source: Infancy. 2026 Jul 18;31(4):e70108. doi: 10.1111/infa.70108 (PMC13380311; doi:10.1111/infa.70108)
Supplement: Supplementary file 1 — Supporting Information S1 [file INFA-31-0-s001.docx]

Supplementary Data for

**Brain activity of young children in Bangladesh is associated with biopsychosocial conditions at the individual-, family-, and household-level**

**Supplementary Methods**

**EEG data processing**

A schematic of the data processing pipeline is provided in Supplementary Figure 1. Data were first referenced to the average of electrodes TP9/10 for data collected at month 3. These electrodes were selected to approximate a linked mastoids reference. Data were instead referenced to electrode Pz for data collected at month 12, as the data quality at electrodes TP9/10 was too poor to use as a reference. We identified excessively noisy channels by visual inspection to identify electrodes that showed persistent, large amplitude artefacts or evidence of a poor or absent connection with the scalp using the conductive gel (mean noisy channels per participant was 1.2, median = 1, range 0-6 for data collected at month 3) and we reconstructed data from these channels at a later step using spherical spline interpolation. There were higher numbers of noisy electrodes for data collected at month 12 due to equipment wear and increased fussiness of the older children (mean = 3.1, median = 2, range 0-11). Data were then high-pass filtered at 1 Hz (EEGLab Basic FIR Filter New, zero-phase, finite impulse response, -6 dB cutoff frequency 0.5 Hz, transition bandwidth 1 Hz) and then low-pass filtered at 30 Hz (same filter type as above, −6 dB cutoff frequency 33.75 Hz, transition bandwidth 7.5 Hz). Data were then segmented into 120-second epochs beginning from the onset of the visual Gabor display. Identified noisy channels were then interpolated using spherical spline interpolation.

Data were then segmented into two-second epochs with 50% overlap between successive epochs. For data collected at month 3 epochs with amplitudes exceeding ±150 µV at any of the channels Fz, F3, F4, FC1, FC2, Cz, C3, C4, CP1, CP2, Pz, P3, P4, Oz, O1, or O2 (i.e., the electrodes used for measurements in defined frequency bands) were removed. For data collected at month 12 epochs with amplitudes exceeding ±150 µV at any of the channels Fz, FC1, FC2, Cz, C3, or C4 (i.e., the electrodes used for month 12 measurements) were removed. Twenty-seven participant-datasets were excluded from group-level analysis for the month 3 data due to insufficient numbers of epochs (less than 58 segments remaining after artefact rejection). 148 datasets were excluded from data collected at month 12. We then converted data into frequency domain representations using Fast Fourier transforms (FFTs; frequency resolution 0.5 Hz, Hann taper).

**Supplementary Figure 1**. Flowchart depicting each EEG data processing step in order of completion. For further details regarding each processing step and differences between the Month 3 and Month 12 datasets, please see the text description in the Supplementary Methods.

| **Supplemental Table 1:** Concurrent associations between biopsychosocial characteristics and EEG outcomes at 11 months child's age | | | | | | | | | | | | | | | | | | | | |
| --- | --- | --- | --- | --- | --- | --- | --- | --- | --- | --- | --- | --- | --- | --- | --- | --- | --- | --- | --- | --- |
|  |  |  |  |  |  |  |  |  |  |  |  |  |  |  |  |  |  |  |  |  |
| **Biopsychosocial characteristics (at 11 months of age)** |  | **Mu alpha (at 11 months of age)** | | |  | **Posterior alpha (at 11 months of age)** | | |  | **Beta (at 11 months of age)** | | |  | **Delta (at 11 months of age)** | | |  | **Theta (at 11 months of age)** | | |
|  | **Model** | **Unstandardized estimate of association (95% CI)** | **P-value** | **Standardized estimate of association (95% CI)** |  | **Unstandardized estimate of association (95% CI)** | **P-value** | **Standardized estimate of association (95% CI)** |  | **Unstandardized estimate of association (95% CI)** | **P-value** | **Standardized estimate of association (95% CI)** |  | **Unstandardized estimate of association (95% CI)** | **P-value** | **Standardized estimate of association (95% CI)** |  | **Unstandardized estimate of association (95% CI)** | **P-value** | **Standardized estimate of association (95% CI)** |
| Maternal depression^3^ | Unadjusted^1^ | 0(-0.009,0.008) | 0.957 |  |  | 0.001(-0.005,0.007) | 0.701 |  |  | -0.001(-0.004,0.002) | 0.450 |  |  | -0.013(-0.064,0.038) | 0.620 |  |  | -0.006(-0.03,0.019) | 0.646 |  |
|  | Adjusted^2^ | 0(-0.01,0.009) | 0.967 | -0.002 (-0.012, 0.007) |  | -0.001(-0.008,0.006) | 0.761 | -0.017 (-0.024, -0.010) |  | -0.003(-0.006,0.001) | 0.115 | -0.091 ( -0.094, -0.088) |  | -0.013(-0.067,0.042) | 0.641 | -0.027 ( -0.082, 0.028) |  | -0.004(-0.031,0.023) | 0.746 | -0.019 (-0.046, 0.008) |
| Household food secure status^4^ | Unadjusted^1^ | -0.019(-0.22,0.182) | 0.851 |  |  | -0.094(-0.237,0.05) | 0.202 |  |  | -0.05(-0.115,0.015) | 0.133 |  |  | -0.396(-1.555,0.763) | 0.502 |  |  | -0.33(-0.893,0.232) | 0.249 |  |
|  | Adjusted^2^ | -0.055(-0.281,0.17) | 0.630 | -0.028(-0.254, 0.198) |  | -0.145(-0.298,0.009) | 0.065 | -0.107(-0.261, 0.046) |  | -0.069(-0.145,0.007) | 0.076 | -0.106 (-0.182, -0.03) |  | -0.688(-1.966,0.59) | 0.290 | -0.063 (-1.341, 1.215) |  | -0.212(-0.845,0.42) | 0.510 | -0.040 (-0.672, 0.593) |
| Psychosocial stimulation^5^ | Unadjusted^1^ | 0.001(-0.011,0.013) | 0.851 |  |  | 0.002(-0.007,0.01) | 0.687 |  |  | 0(-0.004,0.004) | 0.823 |  |  | 0.020(-0.049,0.09) | 0.567 |  |  | -0.003(-0.037,0.031) | 0.870 |  |
|  | Adjusted^2^ | -0.002(-0.016,0.011) | 0.727 | -0.020 (-0.034, -0.007) |  | -0.006(-0.015,0.003) | 0.220 | -0.071 (-0.081, -0.062) |  | 0.001(-0.003,0.006) | 0.647 | 0.027 (0.023, 0.032) |  | 0.017(-0.059,0.092) | 0.668 | 0.026 (-0.050, 0.102) |  | -0.02(-0.057,0.018) | 0.298 | -0.063 (-0.100, -0.026) |
| Hemoglobin (g/l) | Unadjusted^1^ | -0.001(-0.01,0.008) | 0.771 |  |  | -0.003(-0.009,0.003) | 0.322 |  |  | -0.002(-0.005,0.001) | 0.158 |  |  | -0.039(-0.089,0.011) | 0.129 |  |  | -0.003(-0.028,0.021) | 0.783 |  |
|  | Adjusted^2^ | -0.004(-0.014,0.007) | 0.509 | -0.043 (-0.054, -0.033) |  | -0.006(-0.014,0.001) | 0.093 | -0.11(-0.117, -0.103) |  | -0.001(-0.005,0.002) | 0.419 | -0.05442(-0.058, -0.051) |  | -0.034(-0.095,0.027) | 0.277 | -0.073 (-0.134, -0.012) |  | -0.011(-0.041,0.02) | 0.491 | -0.047(-0.077, -0.017) |
| Log_10_ Ferritin (g/ml) | Unadjusted^1^ | -0.023(-0.128,0.082) | 0.667 |  |  | 0.016(-0.056,0.089) | 0.656 |  |  | -0.014(-0.048,0.021) | 0.445 |  |  | -0.427(-1.007,0.153) | 0.149 |  |  | 0(-0.286,0.287) | 0.998 |  |
|  | Adjusted^2^ | 0.013(-0.116,0.143) | 0.839 | 0.014 (-0.115, 0.143) |  | 0.069(-0.019,0.156) | 0.126 | 0.105 (0.017, 0.192) |  | -0.01(-0.053,0.034) | 0.656 | -0.031 (-0.075, 0.012) |  | -0.211(-0.941,0.52) | 0.571 | -0.04 (-0.771, 0.691) |  | 0.09(-0.272,0.452) | 0.626 | 0.035 (-0.327, 0.397) |
| Log_10_ C-reactive protein (mg/l) | Unadjusted^1^ | -0.061(-0.127,0.005) | 0.071 |  |  | -0.016(-0.062,0.029) | 0.476 |  |  | -0.004(-0.026,0.018) | 0.744 |  |  | -0.266(-0.633,0.1) | 0.154 |  |  | -0.086(-0.267,0.094) | 0.347 |  |
|  | Adjusted^2^ | -0.058(-0.127,0.011) | 0.100 | -0.095(-0.164, -0.025) |  | -0.023(-0.07,0.024) | 0.329 | -0.056 (-0.103, -0.009) |  | -0.001(-0.024,0.023) | 0.958 | -0.003 (-0.026, 0.020) |  | -0.247(-0.639,0.146) | 0.217 | -0.073 (-0.465, 0.320) |  | -0.119(-0.313,0.075) | 0.228 | -0.072(-0.266, 0.122) |
| Weight-for-age z scores | Unadjusted^1^ | 0.049(-0.032,0.13) | 0.233 |  |  | 0.031(-0.027,0.089) | 0.294 |  |  | -0.007(-0.034,0.019) | 0.581 |  |  | 0.327(-0.141,0.794) | 0.170 |  |  | -0.004(-0.231,0.223) | 0.971 |  |
|  | Adjusted^2^ | -0.051(-0.189,0.086) | 0.463 | -0.063 (-0.201, 0.075) |  | -0.046(-0.14,0.048) | 0.333 | -0.083 (-0.176, 0.011) |  | -0.053(-0.099,-0.007) | 0.025 | -0.198 (-0.245, -0.152) |  | 0.015(-0.765,0.796) | 0.969 | 0.003 (-0.777, 0.784) |  | 0.089(-0.298,0.475) | 0.652 | 0.040 (-0.346, 0.426) |
| Length-for-age z scores | Unadjusted^1^ | 0.107(0.03,0.185) | 0.007 |  |  | 0.082(0.026,0.137) | 0.004 |  |  | 0.018(-0.008,0.044) | 0.174 |  |  | 0.399(-0.052,0.851) | 0.082 |  |  | 0.053(-0.167,0.272) | 0.636 |  |
|  | Adjusted^2^ | 0.153(0.023,0.282) | 0.021 | 0.197 (0.068, 0.326) |  | 0.112(0.024,0.2) | 0.013 | 0.211 (0.123, 0.299) |  | 0.046(0.002,0.089) | 0.040 | 0.179 (0.135, 0.222) |  | 0.356(-0.375,1.086) | 0.339 | 0.083 (-0.647, 0.814) |  | -0.013(-0.375,0.349) | 0.944 | -0.006 (-0.368, 0.356) |
|  |  |  |  |  |  |  |  |  |  |  |  |  |  |  |  |  |  |  |  |  |
| ^1^Estimates of association were derived using linear regression models, adjusted for number of children under 5 years of age living in the household, parental education, wealth, child sex, religion, union, and intervention group assignment | | | | | | | | | | | | | | | | | | | | |
| ^2^Estimates of association were derived using linear regression models which included all biopsychosocial characteristics, adjusted for number of children under 5 years of age living in the household, parental education, wealth, child sex, religion, union, and intervention group assignment | | | | | | | | | | | | | | | | | | | | |
| ^3^Measured using CES-D (Score range 0 - 35) | | |  |  |  |  |  |  |  |  |  |  |  |  |  |  |  |  |  |  |
| ^4^Measured using the HFIAS (0 indicating presence of food insecurity and 1 indicating presence of food security) | | | | | | | | |  |  |  |  |  |  |  |  |  |  |  |  |
| ^5^Measured using the FCI (Score range 0 - 42) | | |  |  |  |  |  |  |  |  |  |  |  |  |  |  |  |  |  |  |

| **Supplemental Table 2:** Concurrent associations between biopsychosocial characteristics and EEG outcomes at 20 months child's age | | | | | | | | | | | | | | | | | | | | |
| --- | --- | --- | --- | --- | --- | --- | --- | --- | --- | --- | --- | --- | --- | --- | --- | --- | --- | --- | --- | --- |
|  |  |  |  |  |  |  |  |  |  |  |  |  |  |  |  |  |  |  |  |  |
| **Biopsychosocial characteristics (at 20 months of age)** |  | **Mu alpha (at 20 months of age)** | | |  | **Posterior alpha (at 20 months of age)** | | |  | **Beta (at 20 months of age)** | | |  | **Delta (at 20 months of age)** | | |  | **Theta (at 20 months of age)** | | |
|  | **Model** | **Unstandardized estimate of association (95% CI)** | **P-value** | **Standardized estimate of association (95% CI)** |  | **Unstandardized estimate of association (95% CI)** | **P-value** | **Standardized estimate of association (95% CI)** |  | **Unstandardized estimate of association (95% CI)** | **P-value** | **Standardized estimate of association (95% CI)** |  | **Unstandardized estimate of association (95% CI)** | **P-value** | **Standardized estimate of association (95% CI)** |  | **Unstandardized estimate of association (95% CI)** | **P-value** | **Standardized estimate of association (95% CI)** |
| Maternal depression^3^ | Unadjusted^1^ | 0(-0.004,0.005) | 0.859 |  |  | 1.616(-0.001,0.009) | 0.108 |  |  | 0.714(-0.001,0.002) | 0.476 |  |  | -0.472(-0.009,0.005) | 0.637 |  |  | -0.535(-0.006,0.003) | 0.593 |  |
|  | Adjusted^2^ | 0.001(-0.004,0.005) | 0.739 | 0.020 (0.016, 0.025) |  | 0.003(-0.003,0.009) | 0.342 | 0.086 (0.080, 0.092) |  | 0.001(-0.001,0.002) | 0.471 | 0.044 (0.042, 0.046) |  | -0.005(-0.013,0.003) | 0.249 | -0.070 (-0.077, -0.062) |  | -0.002(-0.007,0.003) | 0.410 | -0.049 (-0.054, -0.044) |
| Household food secure status^4^ | Unadjusted^1^ | -0.013(-0.098,0.071) | 0.755 |  |  | -0.051(-0.158,0.055) | 0.340 |  |  | -0.024(-0.059,0.012) | 0.194 |  |  | -0.151(-0.289,-0.013) | 0.032 |  |  | -0.046(-0.138,0.047) | 0.330 |  |
|  | Adjusted^2^ | 0.034(-0.066,0.134) | 0.501 | 0.042 (-0.057, 0.142) |  | -0.031(-0.165,0.104) | 0.655 | -0.043(-0.177, 0.092) |  | 0.025(-0.012,0.062) | 0.184 | 0.084 (0.047, 0.121) |  | -0.236(-0.405,-0.068) | 0.006^6^ | -0.171489 (-0.340, -0.003) |  | -0.034(-0.144,0.075) | 0.539 | -0.037(-0.147, 0.072) |
| Psychosocial stimulation^5^ | Unadjusted^1^ | -0.004(-0.009,0.001) | 0.089 |  |  | -0.004(-0.011,0.002) | 0.170 |  |  | -0.001(-0.003,0.001) | 0.583 |  |  | 0.002(-0.006,0.01) | 0.578 |  |  | -0.003(-0.008,0.003) | 0.334 |  |
|  | Adjusted^2^ | -0.005(-0.011,0) | 0.041 | -0.129 (-0.134, -0.124) |  | -0.003(-0.01,0.004) | 0.439 | -0.073(-0.081, -0.066) |  | 0(-0.002,0.002) | 0.702 | 0.024 (0.022, 0.026) |  | 0.005(-0.004,0.014) | 0.266 | 0.069 (0.061, 0.078) |  | -0.005(-0.01,0.001) | 0.109 | -0.098(-0.104, -0.092) |
| Hemoglobin (g/l) | Unadjusted^1^ | 0.003(-0.001,0.006) | 0.143 |  |  | 0.004(-0.001,0.009) | 0.097 |  |  | -0.001(-0.002,0.001) | 0.452 |  |  | 0.002(-0.004,0.008) | 0.557 |  |  | 0.004(0.001,0.008) | 0.021 |  |
|  | Adjusted^2^ | 0.004(-0.001,0.008) | 0.145 | 0.111 (0.106, 0.115) |  | 0.004(-0.002,0.011) | 0.172 | 0.156 (0.149, 0.162) |  | 0(-0.002,0.001) | 0.723 | -0.027 (-0.029, -0.025) |  | 0.002(-0.006,0.01) | 0.693 | 0.030 (0.022, 0.038) |  | 0.004(-0.001,0.01) | 0.091 | 0.125 (0.119, 0.130) |
| Log_10_ Ferritin (g/ml) | Unadjusted^1^ | 0.025(-0.022,0.072) | 0.290 |  |  | 0.018(-0.045,0.081) | 0.567 |  |  | -0.005(-0.023,0.014) | 0.619 |  |  | 0.039(-0.04,0.118) | 0.334 |  |  | 0.079(0.028,0.13) | 0.003 |  |
|  | Adjusted^2^ | -0.005(-0.07,0.059) | 0.870 | -0.013 (-0.077, 0.052) |  | -0.009(-0.093,0.075) | 0.835 | -0.023 (-0.107, 0.061) |  | 0.001(-0.023,0.025) | 0.920 | 0.008 (-0.016, 0.032) |  | 0.01(-0.099,0.119) | 0.861 | 0.014 (-0.095, 0.123) |  | 0.03(-0.041,0.101) | 0.407 | 0.064 (-0.007, 0.135) |
| Log_10_ C-reactive protein (mg/l) | Unadjusted^1^ | 0.012(-0.016,0.04) | 0.409 |  |  | -0.027(-0.065,0.012) | 0.179 |  |  | -0.004(-0.015,0.007) | 0.435 |  |  | 0(-0.047,0.048) | 0.988 |  |  | 0.031(0,0.062) | 0.048 |  |
|  | Adjusted^2^ | 0.011(-0.019,0.041) | 0.478 | 0.045 (0.014, 0.075) |  | -0.02(-0.062,0.021) | 0.337 | -0.086 (-0.128, -0.044) |  | -0.007(-0.018,0.004) | 0.234 | -0.075 (-0.086, -0.064) |  | -0.016(-0.068,0.035) | 0.530 | -0.039 (-0.091, 0.012) |  | 0.021(-0.012,0.055) | 0.217 | 0.075 (0.042, 0.109) |
| Weight-for-age z scores | Unadjusted^1^ | -0.019(-0.054,0.016) | 0.280 |  |  | 0.006(-0.037,0.048) | 0.799 |  |  | -0.006(-0.02,0.009) | 0.456 |  |  | -0.077(-0.133,-0.02) | 0.008 |  |  | -0.055(-0.092,-0.017) | 0.004 |  |
|  | Adjusted^2^ | -0.004(-0.06,0.052) | 0.891 | -0.012 (-0.069, 0.044) |  | -0.002(-0.078,0.073) | 0.954 | -0.008 (-0.083, 0.068) |  | -0.012(-0.033,0.009) | 0.253 | -0.102 (-0.123, -0.081) |  | 0.014(-0.082,0.109) | 0.777 | 0.025 (-0.070, 0.120) |  | 0.001(-0.061,0.063) | 0.969 | 0.003 (-0.059, 0.065) |
| Length-for-age z scores | Unadjusted^1^ | -0.026(-0.063,0.011) | 0.163 |  |  | 0.015(-0.031,0.061) | 0.522 |  |  | -0.004(-0.02,0.011) | 0.600 |  |  | -0.103(-0.162,-0.043) | 0.001 |  |  | -0.069(-0.109,-0.029) | 0.001 |  |
|  | Adjusted^2^ | -0.027(-0.087,0.032) | 0.365 | -0.080 (-0.139, -0.020) |  | -0.013(-0.091,0.066) | 0.748 | -0.041 (-0.119, 0.038) |  | -0.004(-0.026,0.018) | 0.719 | -0.032 (-0.054, -0.010) |  | -0.11(-0.211,-0.01) | 0.032 | -0.187428 (-0.288, -0.087) |  | -0.071(-0.136,-0.006) | 0.033 | -0.183 (-0.248, -0.117) |
|  |  |  |  |  |  |  |  |  |  |  |  |  |  |  |  |  |  |  |  |  |
| ^1^Estimates of association were derived using linear regression models, adjusted for number of children under 5 years of age living in the household, parental education, wealth, child sex, religion, union, and intervention group assignment | | | | | | | | | | | | | | | | | | | | |
| ^2^Estimates of association were derived using linear regression models which included all biopsychosocial characteristics, adjusted for number of children under 5 years of age living in the household, parental education, wealth, child sex, religion, union, and intervention group assignment | | | | | | | | | | | | | | | | | | | | |
| ^3^Measured using CES-D (Score range 0 - 35) | | |  |  |  |  |  |  |  |  |  |  |  |  |  |  |  |  |  |  |
| ^4^Measured using the HFIAS (0 indicating presence of food insecurity and 1 indicating presence of food security) | | | | | | | | |  |  |  |  |  |  |  |  |  |  |  |  |
| ^5^Measured using the FCI (Score range 0 - 42) | | |  |  |  |  |  |  |  |  |  |  |  |  |  |  |  |  |  |  |
| ^6^Q = 0.048. All other Q-values >0.05. Q refers to the false discovery rate adjusted p value, by model." | | | | | | | | | | | | | | | | | | | | |

| **Supplemental Table 3:** Lagged relationship between biopsychosocial characteristics at 8 months and EEG outcomes at 11 months child's age | | | | | | | | | | | | | | | | | | | | |
| --- | --- | --- | --- | --- | --- | --- | --- | --- | --- | --- | --- | --- | --- | --- | --- | --- | --- | --- | --- | --- |
|  |  |  |  |  |  |  |  |  |  |  |  |  |  |  |  |  |  |  |  |  |
| **Biopsychosocial characteristics (at 8 months of age)** |  | **Mu alpha (at 11 months of age)** | | |  | **Posterior alpha (at 11 months of age)** | | |  | **Beta (at 11 months of age)** | | |  | **Delta (at 11 months of age)** | | |  | **Theta (at 11 months of age)** | | |
|  | **Model** | **Unstandardized estimate of association (95% CI)** | **P-value** | **Standardized estimate of association (95% CI)** |  | **Unstandardized estimate of association (95% CI)** | **P-value** | **Standardized estimate of association (95% CI)** |  | **Unstandardized estimate of association (95% CI)** | **P-value** | **Standardized estimate of association (95% CI)** |  | **Unstandardized estimate of association (95% CI)** | **P-value** | **Standardized estimate of association (95% CI)** |  | **Unstandardized estimate of association (95% CI)** | **P-value** | **Standardized estimate of association (95% CI)** |
| Maternal depression^3^ | Unadjusted^1^ | 0.004(-0.006,0.014) | 0.397 |  |  | 0.008(0.001,0.015) | 0.027 |  |  | -0.001(-0.004,0.003) | 0.739 |  |  | -0.005(-0.063,0.053) | 0.866 |  |  | -0.016(-0.044,0.012) | 0.271 |  |
|  | Adjusted^2^ | 0.002(-0.009,0.013) | 0.696 | 0.02(0.009,0.031) |  | 0.007(-0.001,0.014) | 0.076 | 0.091(0.083,0.098) |  | -0.002(-0.005,0.002) | 0.362 | -0.048(-0.051,-0.044) |  | -0.004(-0.066,0.058) | 0.894 | -0.007(-0.069,0.055) |  | -0.021(-0.051,0.009) | 0.166 | -0.073(-0.103,-0.043) |
| Household food secure status^4^ | Unadjusted^1^ | -0.054(-0.275,0.166) | 0.628 |  |  | -0.102(-0.26,0.056) | 0.207 |  |  | -0.048(-0.121,0.024) | 0.191 |  |  | 0.426(-0.852,1.704) | 0.512 |  |  | -0.15(-0.769,0.469) | 0.635 |  |
|  | Adjusted^2^ | 0.009(-0.223,0.241) | 0.940 | 0.004(-0.228,0.236) |  | -0.052(-0.218,0.113) | 0.535 | -0.034(-0.199,0.132) |  | -0.051(-0.128,0.026) | 0.191 | -0.073(-0.15,0.004) |  | 0.502(-0.851,1.856) | 0.466 | 0.041(-1.313,1.395) |  | -0.2(-0.856,0.456) | 0.549 | -0.034(-0.69,0.622) |
| Psychosocial stimulation^5^ | Unadjusted^1^ | 0.002(-0.01,0.014) | 0.717 |  |  | 0.002(-0.007,0.01) | 0.731 |  |  | 0.001(-0.003,0.005) | 0.613 |  |  | -0.012(-0.081,0.058) | 0.742 |  |  | 0.017(-0.017,0.05) | 0.333 |  |
|  | Adjusted^2^ | 0(-0.013,0.012) | 0.956 | -0.003(-0.015,0.009) |  | 0.002(-0.007,0.01) | 0.723 | 0.019(0.01,0.028) |  | 0.001(-0.003,0.005) | 0.745 | 0.018(0.014,0.022) |  | -0.014(-0.086,0.058) | 0.698 | -0.021(-0.093,0.051) |  | 0.013(-0.022,0.048) | 0.474 | 0.039(0.004,0.074) |
| Hemoglobin (g/l) | Unadjusted^1^ | 0(-0.008,0.009) | 0.965 |  |  | -0.001(-0.007,0.005) | 0.710 |  |  | -0.001(-0.004,0.002) | 0.590 |  |  | 0.015(-0.034,0.065) | 0.546 |  |  | 0.005(-0.019,0.029) | 0.679 |  |
|  | Adjusted^2^ | 0.001(-0.01,0.011) | 0.911 | 0.007(-0.004,0.017) |  | -0.002(-0.009,0.005) | 0.558 | -0.034(-0.041,-0.027) |  | -0.001(-0.005,0.002) | 0.482 | -0.042(-0.045,-0.038) |  | 0.022(-0.037,0.081) | 0.460 | 0.044(-0.015,0.103) |  | 0.006(-0.022,0.035) | 0.674 | 0.025(-0.003,0.054) |
| Log_10_ Ferritin (g/ml) | Unadjusted^1^ | 0.021(-0.084,0.126) | 0.698 |  |  | -0.021(-0.096,0.054) | 0.582 |  |  | -0.018(-0.053,0.016) | 0.297 |  |  | -0.053(-0.661,0.555) | 0.864 |  |  | 0.084(-0.211,0.379) | 0.577 |  |
|  | Adjusted^2^ | 0.014(-0.11,0.138) | 0.821 | 0.014(-0.11,0.138) |  | 0(-0.088,0.089) | 0.995 | 0(-0.088,0.089) |  | -0.01(-0.052,0.031) | 0.620 | -0.031(-0.072,0.011) |  | -0.166(-0.891,0.559) | 0.653 | -0.028(-0.753,0.697) |  | 0.054(-0.297,0.405) | 0.763 | 0.019(-0.332,0.37) |
| Log_10_ C-reactive protein (mg/l) | Unadjusted^1^ | 0.025(-0.033,0.083) |  |  |  | -0.006(-0.047,0.036) |  |  |  | -0.011(-0.03,0.008) |  |  |  | 0.013(-0.324,0.351) | 0.938 |  |  | 0.015(-0.148,0.178) | 0.857 |  |
|  | Adjusted^2^ | 0.03(-0.035,0.094) | 0.368 | 0.05(-0.014,0.114) |  | -0.007(-0.053,0.038) | 0.753 | -0.017(-0.063,0.028) |  | -0.011(-0.032,0.01) | 0.308 | -0.058(-0.079,-0.036) |  | 0.067(-0.308,0.442) | 0.727 | 0.02(-0.355,0.395) |  | 0.029(-0.153,0.211) | 0.753 | 0.018(-0.164,0.2) |
| Weight-for-age z scores | Unadjusted^1^ | 0.06(-0.02,0.14) | 0.141 |  |  | 0.039(-0.018,0.096) | 0.180 |  |  | -0.006(-0.033,0.02) | 0.638 |  |  | 0.221(-0.241,0.684) | 0.347 |  |  | 0.044(-0.18,0.268) | 0.701 |  |
|  | Adjusted^2^ | -0.024(-0.136,0.088) | 0.669 | -0.03(-0.142,0.082) |  | -0.006(-0.086,0.073) | 0.873 | -0.011(-0.091,0.069) |  | -0.028(-0.065,0.009) | 0.143 | -0.105(-0.142,-0.068) |  | 0.147(-0.506,0.8) | 0.659 | 0.032(-0.621,0.685) |  | -0.029(-0.346,0.288) | 0.858 | -0.013(-0.33,0.304) |
| Length-for-age z scores | Unadjusted^1^ | 0.108(0.028,0.188) | 0.008 |  |  | 0.062(0.004,0.119) | 0.036 |  |  | 0.008(-0.018,0.035) | 0.532 |  |  | 0.176(-0.291,0.643) | 0.459 |  |  | 0.037(-0.19,0.263) | 0.751 |  |
|  | Adjusted^2^ | 0.126(0.015,0.237) | 0.026 | 0.155(0.044,0.266) |  | 0.065(-0.014,0.144) | 0.107 | 0.111(0.032,0.19) |  | 0.028(-0.009,0.065) | 0.137 | 0.106(0.069,0.142) |  | 0.032(-0.615,0.68) | 0.922 | 0.007(-0.64,0.654) |  | 0.054(-0.26,0.368) | 0.734 | 0.024(-0.29,0.338) |
|  |  |  |  |  |  |  |  |  |  |  |  |  |  |  |  |  |  |  |  |  |
| ^1^Estimates of association were derived using linear regression models, adjusted for number of children under 5 years of age living in the household, parental education, wealth, child sex, religion, union, and intervention group assignment | | | | | | | | | | | | | | | | | | | | |
| ^2^Estimates of association were derived using linear regression models which included all biopsychosocial characteristics, adjusted for number of children under 5 years of age living in the household, parental education, wealth, child sex, religion, union, and intervention group assignment | | | | | | | | | | | | | | | | | | | | |
| ^3^Measured using CES-D (Score range 0 - 35) | | |  |  |  |  |  |  |  |  |  |  |  |  |  |  |  |  |  |  |
| ^4^Measured using the HFIAS (0 indicating presence of food insecurity and 1 indicating presence of food security) | | | | | | | | | | |  |  |  |  |  |  |  |  |  |  |
| ^5^Measured using the FCI (Score range 0 - 42) | | |  |  |  |  |  |  |  |  |  |  |  |  |  |  |  |  |  |  |

| **Supplemental Table 4:** Lagged relationship between biopsychosocial characteristics at 11 months and EEG outcomes at 20 months child's age | | | | | | | | | | | | | | | | | | | | |
| --- | --- | --- | --- | --- | --- | --- | --- | --- | --- | --- | --- | --- | --- | --- | --- | --- | --- | --- | --- | --- |
|  |  |  |  |  |  |  |  |  |  |  |  |  |  |  |  |  |  |  |  |  |
| **Biopsychosocial characteristics (at 11 months of age)** |  | **Mu alpha (at 20 months of age)** | | |  | **Posterior alpha (at 20 months of age)** | | |  | **Beta (at 20 months of age)** | | |  | **Delta (at 20 months of age)** | | |  | **Theta (at 20 months of age)** | | |
|  | **Model** | **Unstandardized estimate of association (95% CI)** | **P-value** | **Standardized estimate of association (95% CI)** |  | **Unstandardized estimate of association (95% CI)** | **P-value** | **Standardized estimate of association (95% CI)** |  | **Unstandardized estimate of association (95% CI)** | **P-value** | **Standardized estimate of association (95% CI)** |  | **Unstandardized estimate of association (95% CI)** | **P-value** | **Standardized estimate of association (95% CI)** |  | **Unstandardized estimate of association (95% CI)** | **P-value** | **Standardized estimate of association (95% CI)** |
| Maternal depression^3^ | Unadjusted^1^ | 0.004(0,0.008) | 0.043 |  |  | 0.005(0,0.011) | 0.072 |  |  | 0.001(-0.001,0.003) | 0.280 |  |  | 0.003(-0.004,0.01) | 0.400 |  |  | 0(-0.004,0.005) | 0.896 |  |
|  | Adjusted^2^ | 0.004(0,0.008) | 0.071 | 0.101 (0.096, 0.105) |  | 0.005(-0.001,0.011) | 0.111 | 0.129 (0.123, 0.35) |  | 0.001(-0.001,0.003) | 0.255 | 0.064 (0.062, 0.066) |  | 0.004(-0.003,0.011) | 0.258 | 0.063 (0.056, 0.070) |  | 0(-0.005,0.005) | 0.945 | 0.004 (-0.001, 0.008) |
| Household food secure status^4^ | Unadjusted^1^ | 0.027(-0.071,0.124) | 0.594 |  |  | -0.029(-0.16,0.101) | 0.659 |  |  | -0.003(-0.042,0.037) | 0.892 |  |  | -0.071(-0.231,0.09) | 0.388 |  |  | 0.037(-0.07,0.144) | 0.502 |  |
|  | Adjusted^2^ | 0.043(-0.058,0.145) | 0.403 | 0.048 ( -0.054, 0.149) |  | -0.015(-0.153,0.122) | 0.825 | -0.019 (-0.156, 0.119) |  | 0.003(-0.038,0.044) | 0.880 | 0.009 (-0.032, 0.050) |  | -0.031(-0.197,0.134) | 0.712 | -0.021 (-0.187, 0.144) |  | 0.036(-0.075,0.148) | 0.521 | 0.037 (-0.075, 0.148) |
| Psychosocial stimulation^5^ | Unadjusted^1^ | -0.003(-0.008,0.002) | 0.256 |  |  | -0.004(-0.01,0.002) | 0.228 |  |  | -0.003(-0.005,-0.001) | 0.005 |  |  | 0.001(-0.007,0.009) | 0.808 |  |  | -0.004(-0.01,0.001) | 0.144 |  |
|  | Adjusted^2^ | -0.002(-0.007,0.003) | 0.412 | -0.046 (-0.051, -0.040) |  | -0.004(-0.01,0.003) | 0.286 | -0.090 (-0.097, -0.083) |  | -0.003(-0.005,-0.001) | 0.013 | -0.140 (-0.143, -0.138) |  | 0.003(-0.005,0.012) | 0.427 | 0.044 (0.036, 0.053) |  | -0.003(-0.008,0.003) | 0.378 | -0.049 (-0.055, -0.043) |
| Hemoglobin (g/l) | Unadjusted^1^ | 0.003(-0.001,0.007) | 0.095 |  |  | 0(-0.005,0.005) | 0.977 |  |  | 0(-0.001,0.002) | 0.823 |  |  | 0.006(-0.001,0.012) | 0.078 |  |  | 0.003(-0.001,0.007) | 0.185 |  |
|  | Adjusted^2^ | 0.004(-0.001,0.009) | 0.103 | 0.103 (0.098, 0.108) |  | 0.001(-0.006,0.007) | 0.823 | 0.021 (0.014, 0.027) |  | 0(-0.002,0.002) | 0.818 | -0.015 (-0.017, -0.013) |  | 0.005(-0.002,0.013) | 0.183 | 0.084 ( 0.077, 0.092) |  | 0.005(0,0.01) | 0.064 | 0.117 (0.111, 0.122) |
| Log_10_ Ferritin (g/ml) | Unadjusted^1^ | 0.035(-0.008,0.078) | 0.112 |  |  | 0.003(-0.051,0.056) | 0.924 |  |  | 0.005(-0.013,0.024) | 0.555 |  |  | 0.052(-0.019,0.123) | 0.149 |  |  | 0.029(-0.019,0.077) | 0.235 |  |
|  | Adjusted^2^ | 0.007(-0.046,0.059) | 0.809 | 0.016(-0.036, 0.069) |  | -0.012(-0.079,0.056) | 0.736 | -0.034 (-0.102, 0.034) |  | 0.007(-0.015,0.028) | 0.545 | 0.041(0.020, 0.063) |  | 0.031(-0.055,0.117) | 0.481 | 0.048 (-0.038, 0.134) |  | -0.008(-0.066,0.05) | 0.783 | -0.019 (-0.077, 0.040) |
| Log_10_ C-reactive protein (mg/l) | Unadjusted^1^ | 0.017(-0.009,0.042) | 0.204 |  |  | 0.02(-0.014,0.054) | 0.247 |  |  | -0.002(-0.013,0.009) | 0.717 |  |  | -0.005(-0.047,0.037) | 0.811 |  |  | 0.022(-0.007,0.05) | 0.137 |  |
|  | Adjusted^2^ | 0.014(-0.015,0.043) | 0.357 | 0.057 (0.028, 0.086) |  | 0.024(-0.014,0.061) | 0.217 | 0.107 (0.069, 0.144) |  | -0.007(-0.018,0.005) | 0.264 | -0.069 (-0.081, -0.058) |  | -0.009(-0.057,0.038) | 0.702 | -0.024 (-0.071, 0.024) |  | 0.022(-0.01,0.054) | 0.174 | 0.083 (0.051, 0.115) |
| Weight-for-age z scores | Unadjusted^1^ | -0.024(-0.06,0.011) | 0.177 |  |  | 0.016(-0.029,0.061) | 0.483 |  |  | 0(-0.015,0.014) | 0.952 |  |  | -0.069(-0.127,-0.011) | 0.019 |  |  | -0.061(-0.099,-0.023) | 0.002 |  |
|  | Adjusted^2^ | -0.018(-0.068,0.031) | 0.468 | -0.052 (-0.102, -0.003) |  | 0.018(-0.051,0.086) | 0.610 | 0.056 (-0.012, 0.125) |  | 0.001(-0.019,0.021) | 0.913 | 0.008 (-0.012, 0.028) |  | -0.037(-0.117,0.044) | 0.374 | -0.065 (-0.145, 0.016) |  | -0.04(-0.095,0.014) | 0.145 | -0.105 (-0.159, -0.050) |
| Length-for-age z scores | Unadjusted^1^ | -0.02(-0.055,0.015) | 0.258 |  |  | 0.014(-0.031,0.059) | 0.544 |  |  | -0.001(-0.016,0.014) | 0.925 |  |  | -0.001(-0.016,0.014) | 0.925 |  |  | -0.062(-0.1,-0.024) | 0.001 |  |
|  | Adjusted^2^ | -0.014(-0.062,0.033) | 0.548 | -0.043 (-0.090, 0.004) |  | 0.005(-0.06,0.07) | 0.884 | 0.016 (-0.049, 0.082) |  | -0.004(-0.022,0.015) | 0.716 | -0.026 ( -0.045, -0.007) |  | -0.065(-0.142,0.012) | 0.098 | -0.119 (-0.196, -0.043) |  | -0.041(-0.093,0.011) | 0.124 | -0.110 (-0.162, -0.058) |
|  |  |  |  |  |  |  |  |  |  |  |  |  |  |  |  |  |  |  |  |  |
| ^1^Estimates of association were derived using linear regression models, adjusted for number of children under 5 years of age living in the household, parental education, wealth, child sex, religion, union, and intervention group assignment | | | | | | | | | | | | | | | | | | | | |
| ^2^Estimates of association were derived using linear regression models which included all biopsychosocial characteristics, adjusted for number of children under 5 years of age living in the household, parental education, wealth, child sex, religion, union, and intervention group assignment | | | | | | | | | | | | | | | | | | | | |
| ^3^Measured using CES-D (Score range 0 - 35) | | |  |  |  |  |  |  |  |  |  |  |  |  |  |  |  |  |  |  |
| ^4^Measured using the HFIAS (0 indicating presence of food insecurity and 1 indicating presence of food security) | | | | | | | | | |  |  |  |  |  |  |  |  |  |  |  |
| ^5^Measured using the FCI (Score range 0 - 42) | | |  |  |  |  |  |  |  |  |  |  |  |  |  |  |  |  |  |  |

| **Supplemental Table 5:** Lagged relationship between biopsychosocial characteristics at 8 months and EEG outcomes at 20 months child's age | | | | | | | | | | | | | | | | | | | | |
| --- | --- | --- | --- | --- | --- | --- | --- | --- | --- | --- | --- | --- | --- | --- | --- | --- | --- | --- | --- | --- |
|  |  |  |  |  |  |  |  |  |  |  |  |  |  |  |  |  |  |  |  |  |
| **Biopsychosocial characteristics (at 8 months of age)** |  | **Mu alpha (at 20 months of age)** | | |  | **Posterior alpha (at 20 months of age)** | | |  | **Beta (at 20 months of age)** | | |  | **Delta (at 20 months of age)** | | |  | **Theta (at 20 months of age)** | | |
|  | **Model** | **Unstandardized estimate of association (95% CI)** | **P-value** | **Standardized estimate of association (95% CI)** |  | **Unstandardized estimate of association (95% CI)** | **P-value** | **Standardized estimate of association (95% CI)** |  | **Unstandardized estimate of association (95% CI)** | **P-value** | **Standardized estimate of association (95% CI)** |  | **Unstandardized estimate of association (95% CI)** | **P-value** | **Standardized estimate of association (95% CI)** |  | **Unstandardized estimate of association (95% CI)** | **P-value** | **Standardized estimate of association (95% CI)** |
| Maternal depression^3^ | Unadjusted^1^ | 0.002(-0.004,0.008) | 0.493 |  |  | 0.003(-0.007,0.013) | 0.581 |  |  | 0(-0.003,0.003) | 0.954 |  |  | -0.002(-0.012,0.008) | 0.643 |  |  | 0.001(-0.006,0.007) | 0.876 |  |
|  | Adjusted^2^ | 0.002(-0.005,0.008) | 0.600 | 0.049(0.042,0.055) |  | -0.002(-0.014,0.011) | 0.763 | -0.042(-0.055,-0.03) |  | 0.001(-0.002,0.004) | 0.598 | 0.051(0.048,0.054) |  | -0.008(-0.019,0.003) | 0.169 | -0.126(-0.137,-0.115) |  | 0(-0.007,0.007) | 0.981 | -0.002(-0.009,0.005) |
| Household food secure status^4^ | Unadjusted^1^ | -0.029(-0.165,0.108) | 0.678 |  |  | 0.029(-0.166,0.223) | 0.769 |  |  | -0.017(-0.081,0.048) | 0.609 |  |  | -0.197(-0.424,0.03) | 0.089 |  |  | -0.002(-0.158,0.154) | 0.977 |  |
|  | Adjusted^2^ | -0.01(-0.167,0.148) | 0.905 | -0.011(-0.169,0.146) |  | -0.064(-0.332,0.203) | 0.631 | -0.074(-0.342,0.195) |  | 0.041(-0.031,0.113) | 0.262 | 0.11(0.038,0.181) |  | -0.18(-0.446,0.087) | 0.185 | -0.121(-0.388,0.145) |  | 0.042(-0.136,0.22) | 0.639 | 0.043(-0.135,0.221) |
| Psychosocial stimulation^5^ | Unadjusted^1^ | -0.007(-0.014,0.001) | 0.094 |  |  | -0.008(-0.02,0.003) | 0.163 |  |  | -0.002(-0.006,0.002) | 0.293 |  |  | -0.001(-0.014,0.012) | 0.899 |  |  | -0.01(-0.019,-0.002) | 0.021 |  |
|  | Adjusted^2^ | -0.006(-0.014,0.003) | 0.205 | -0.123(-0.131,-0.114) |  | -0.009(-0.024,0.005) | 0.199 | -0.219(-0.233,-0.204) |  | 0(-0.004,0.004) | 0.844 | -0.02(-0.024,-0.016) |  | 0.003(-0.011,0.018) | 0.658 | 0.042(0.027,0.057) |  | -0.01(-0.02,0) | 0.046 | -0.192(-0.202,-0.182) |
| Hemoglobin (g/l) | Unadjusted^1^ | 0.001(-0.004,0.006) | 0.719 |  |  | 0.001(-0.006,0.009) | 0.713 |  |  | -0.002(-0.004,0) | 0.113 |  |  | 0.001(-0.009,0.011) | 0.793 |  |  | 0.002(-0.004,0.008) | 0.562 |  |
|  | Adjusted^2^ | 0.002(-0.005,0.009) | 0.632 | 0.052(0.045,0.059) |  | 0.003(-0.008,0.014) | 0.613 | 0.096(0.085,0.107) |  | -0.002(-0.005,0.001) | 0.195 | -0.149(-0.152,-0.145) |  | 0.011(-0.001,0.023) | 0.080 | 0.188(0.176,0.2) |  | 0.006(-0.002,0.015) | 0.113 | 0.172(0.164,0.18) |
| Log_10_ Ferritin (g/ml) | Unadjusted^1^ | -0.005(-0.073,0.063) | 0.887 |  |  | -0.048(-0.146,0.05) | 0.327 |  |  | -0.017(-0.048,0.014) | 0.275 |  |  | -0.066(-0.184,0.052) | 0.271 |  |  | -0.025(-0.103,0.054) | 0.539 |  |
|  | Adjusted^2^ | -0.011(-0.101,0.079) | 0.802 | -0.029(-0.114,0.061) |  | -0.089(-0.234,0.057) | 0.226 | -0.233(-0.378,-0.088) |  | 0.001(-0.04,0.042) | 0.946 | 0.008(-0.033,0.049) |  | -0.166(-0.318,-0.014) | 0.033 | -0.243(-0.396,-0.091) |  | -0.084(-0.186,0.017) | 0.103 | -0.187(-0.289,-0.086) |
| Log_10_ C-reactive protein (mg/l) | Unadjusted^1^ | 0.025(-0.02,0.07) | 0.279 |  |  | -0.026(-0.094,0.042) | 0.451 |  |  | -0.011(-0.032,0.01) | 0.289 |  |  | 0.045(-0.034,0.123) | 0.262 |  |  | 0.025(-0.027,0.077) | 0.342 |  |
|  | Adjusted^2^ | 0.022(-0.027,0.071) | 0.380 | 0.078(0.030,0.128) |  | -0.014(-0.093,0.065) | 0.729 | -0.051(-0.131,0.029) |  | -0.013(-0.035,0.01) | 0.259 | -0.106(-0.128,-0.084) |  | 0.063(-0.02,0.146) | 0.133 | 0.132(0.049,0.215) |  | 0.026(-0.03,0.081) | 0.362 | 0.081(0.025,0.136) |
| Weight-for-age z scores | Unadjusted^1^ | -0.002(-0.061,0.057) | 0.944 |  |  | 0.043(-0.035,0.122) | 0.275 |  |  | -0.001(-0.029,0.027) | 0.930 |  |  | -0.072(-0.172,0.028) | 0.158 |  |  | -0.062(-0.128,0.004) | 0.067 |  |
|  | Adjusted^2^ | 0.049(-0.059,0.156) | 0.371 | 0.127(0.020,0.234) |  | -0.021(-0.194,0.151) | 0.806 | -0.061(-0.233,0.113) |  | 0.006(-0.043,0.055) | 0.807 | 0.036(-0.013,0.085) |  | -0.015(-0.196,0.167) | 0.873 | -0.022(-0.203,0.159) |  | -0.032(-0.153,0.089) | 0.601 | -0.073(-0.194,0.048) |
| Length-for-age z scores | Unadjusted^1^ | -0.038(-0.093,0.018) | 0.183 |  |  | 0.055(-0.017,0.127) | 0.132 |  |  | 0(-0.027,0.026) | 0.984 |  |  | -0.09(-0.184,0.004) | 0.060 |  |  | -0.067(-0.129,-0.005) | 0.034 |  |
|  | Adjusted^2^ | -0.048(-0.144,0.047) | 0.321 | -0.136(-0.232,-0.041) |  | 0.042(-0.109,0.192) | 0.582 | 0.135(-0.016,0.286) |  | -0.013(-0.056,0.031) | 0.566 | -0.082(-0.126,-0.039) |  | -0.1(-0.262,0.061) | 0.222 | -0.164(-0.326,-0.003) |  | -0.053(-0.161,0.055) | 0.332 | -0.132(-0.24,-0.024) |
|  |  |  |  |  |  |  |  |  |  |  |  |  |  |  |  |  |  |  |  |  |
| ^1^Estimates of association were derived using linear regression models, adjusted for number of children under 5 years of age living in the household, parental education, wealth, child sex, religion, union, and intervention group assignment | | | | | | | | | | | | | | | | | | | | |
| ^2^Estimates of association were derived using linear regression models which included all biopsychosocial characteristics, adjusted for number of children under 5 years of age living in the household, parental education, wealth, child sex, religion, union, and intervention group assignment | | | | | | | | | | | | | | | | | | | | |
| ^3^Measured using CES-D (Score range 0 - 35) | | |  |  |  |  |  |  |  |  |  |  |  |  |  |  |  |  |  |  |
| ^4^Measured using the HFIAS (0 indicating presence of food insecurity and 1 indicating presence of food security) | | | | | | | | | | |  |  |  |  |  |  |  |  |  |  |
| ^5^Measured using the FCI (Score range 0 - 42) | | |  |  |  |  |  |  |  |  |  |  |  |  |  |  |  |  |  |  |
